# Time-dependent unloading effects on muscle and bone and involvement of FNDC5/irisin axis

**DOI:** 10.1038/s41526-023-00251-w

**Published:** 2023-01-19

**Authors:** Lorenzo Sanesi, Giuseppina Storlino, Manuela Dicarlo, Angela Oranger, Roberta Zerlotin, Patrizia Pignataro, Clelia Suriano, Gabriella Guida, Maria Grano, Graziana Colaianni, Silvia Concetta Colucci

**Affiliations:** 1 Department of Translational Biomedicine and Neuroscience, University of Bari, Bari, Italy; 2 Department of Precision and Regenerative Medicine and Ionian Area, University of Bari, Bari, Italy

**Keywords:** Predictive markers, Molecular biology

## Abstract

The identification of biomarkers and countermeasures to prevent the adverse effects on the musculoskeletal system caused by the absence of mechanical loading is the main goal of space biomedical research studies. In this study, we analyzed over 4 weeks of unloading, the modulation in the expression of key proteins in Vastus lateralis, Gastrocnemius and cortical bone in parallel with the modulation of irisin serum levels and its precursor *FNDC5* in skeletal muscle of hind limb unloaded (HU) mice. Here we report that *Atrogin-1* was up-regulated as early as 1- and 2-week of unloading, whereas *Murf-1* at 2- and 3-weeks, along with a marked modulation in the expression of myosin heavy chain isoforms during unloading. Since HU mice showed reduced irisin serum levels at 4-weeks, as well as FNDC5 decrease at 3- and 4-weeks, we treated HU mice with recombinant irisin for 4 weeks, showing that unloading-dependent decline of myosin heavy chain isoforms, *MyHCIIα* and *MyHCIIx*, and the anti-apoptotic factor *Bcl2*, were prevented. In parallel, irisin treatment inhibited the increase of the senescence marker *p53*, and the pro-apoptotic factor *Bax*. Overall, these results suggest that the myokine irisin could be a possible therapy to counteract the musculoskeletal impairment caused by unloading.

## Introduction

Skeletal muscle and bone play a fundamental role in human physiology, enabling locomotion and movement, but in addition to this mechanical role, these two tissues are also important regulators of metabolism throughout the body. Studies in humans and animal models have amply demonstrated that sedentary lifestyle, immobility in bedridden patients, aging, and certain metabolic diseases can cause parallel changes in bone and muscle mass^1,2^. Therefore, increasing attention has been focused on the concept of bone-muscle unit, according to which there is a very close relationship between bone mass/geometry and muscle mass. Intriguingly, recent studies have established that muscle and bone are closely coupled, not only because of their anatomical proximity and mechanical interaction, but also in terms of paracrine and endocrine signals. Under normal physiological conditions, muscle produces myokines in response to the stimulus of contraction and exercise^3,4^. However, unloading conditions can lead to change in the secretion of these molecules and this can contribute to bone loss^2^. One of the most widely used mouse model for studying the effects of weightlessness on the skeletal muscle system is the hindlimb suspension model (HU)^5^. The HU model is an established mouse model for its reproducibility and significant induction of muscle atrophy and bone loss^6,7^. Compared to other murine models of mechanical unloading, the HU model does not require complete immobilization of the hind limbs and this condition makes it possible to effectively mimic what happens during poor mobility and in microgravity conditions. In fact, in this murine model, passive muscular forces persist, and the animals experience a shift of cephalic fluid, a phenomenon that is also characteristic of microgravity conditions. This phenomenon is of crucial importance since reduced skeletal perfusion may induce severe changes in the musculoskeletal system^8^.

Because many diseases show a link between muscle and bone loss, several studies have focused on molecular communication occurring through myokines between these tissues. One of these molecules is the myokine irisin performing a key role in the functioning of the bone-muscle unit^9,10^. Irisin is a hormone-like molecule produced by muscle during physical activity, and in our previous studies, we demonstrated that it increases bone mineral density (BMD) and bone surface in cortical bone of young mice, and it enhances bone geometry by increasing periosteal circumference and polar moment of inertia (pMOI)^11^. Moreover, in HU mice, treatment with recombinant irisin counteracts reduction of trabecular and cortical BMD, bone volume fraction (BV/TV) and prevents muscle wasting and mitochondrial dysfunction in the vastus lateralis^12^.

Compelling evidence is emerging that the myokine irisin can prevent the onset of musculoskeletal atrophy also in humans. Low levels of circulating irisin have been found to be predictive of muscle weakness and atrophy in postmenopausal women^13^. Very recently we investigated a group of patients with Charcot-Marie-Tooth (CMT) disease, a peripheral neuropathy whose primary clinical symptoms are progressive distal weakness, muscle atrophy and 1.5-fold increase in fracture risks. The data showed that circulating irisin levels correlated positively with muscle strength and were predictive of muscle quality and the bone formation marker P1PN, suggesting that irisin may represent a novel biomarker to help monitor the progression of muscle atrophy and bone fragility in patients with CMT^14^.

However, it remains unclear whether the reduction in circulating levels of irisin is a trigger of muscle atrophy or an outcome of muscle inactivity and whether its modulation precedes the resulting negative impact on bone mass.

Therefore, in the present study, we exploited the murine model of unloading, the HU mouse, to decipher the time-differential modulation of key markers of muscle and bone, in relation to the change of circulating irisin and its precursor FNDC5 during 4 weeks of unloading. Specifically, since numerous studies have already addressed the effects on the Soleus muscle^15^, which is known to undergo disuse atrophy in the early stages of HU (1–3 days), in the present study we focused on the effect of unloading on the Vastus Lateralis and Gastrocnemius, two muscles that have been less studied and are affected later by disuse-induced atrophy. Moreover, the rationale of our study is based on the data obtained from our previous study in HU mice treated with irisin^12^, which showed that the Vastus Lateralis, a fast-twitch muscle, was particularly sensitive to the action of irisin in preventing muscle wasting and dysregulation of mitochondrial biogenesis. In parallel, to investigate the effects of the FNDC5/irisin axis in the musculoskeletal system, we examined the effect of recombinant irisin treatment on the bone and muscle cells in vitro, and in HU mice kept in unloading conditions for 4 weeks.

## Results

### Unloading affects muscle morphology, upregulates markers of muscle atrophy and down-regulates fast myosin heavy chain expression

**Fig. 1:**
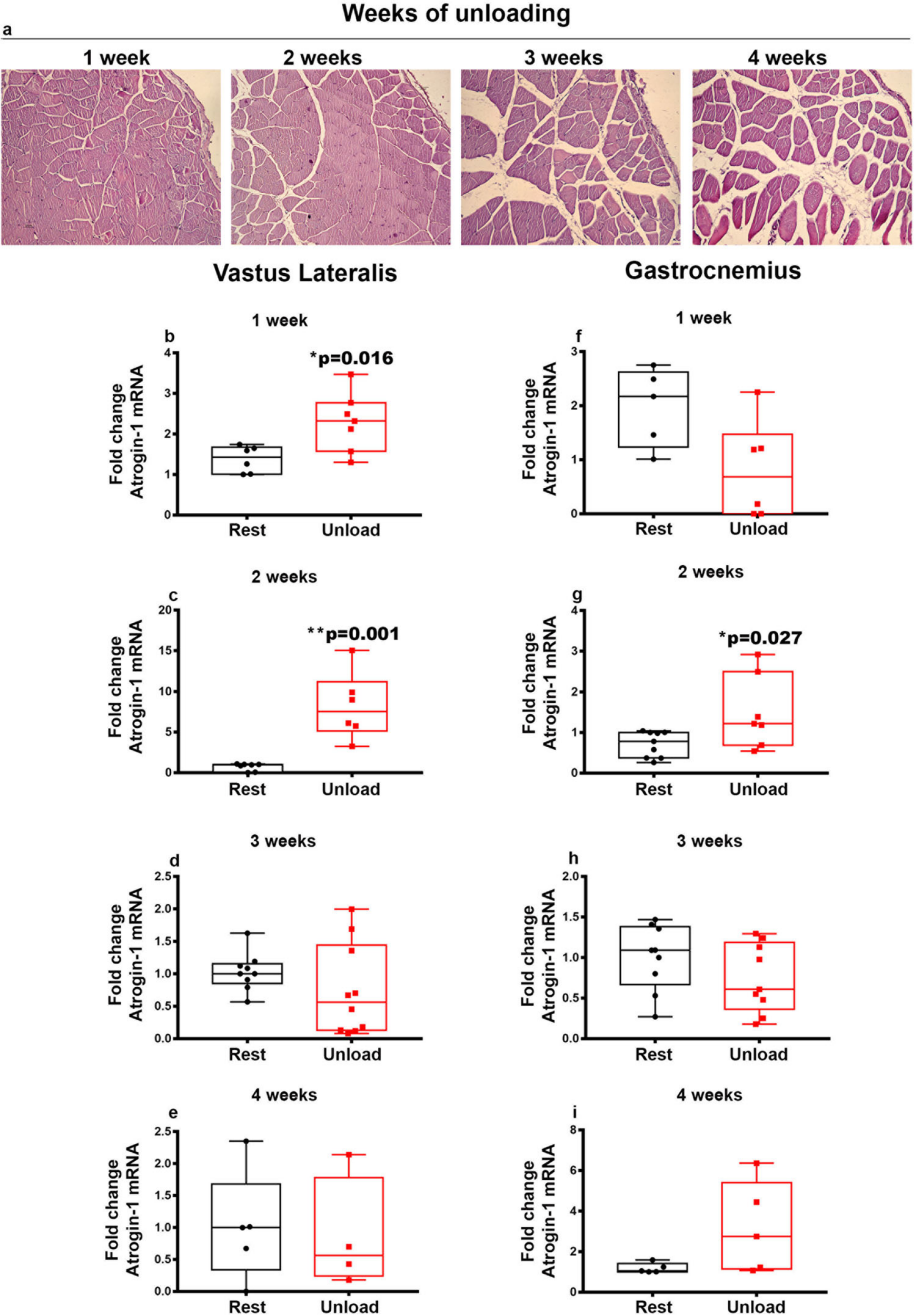
Effect of unloading on muscle morphology and *Atrogin-1* mRNA expression in Vastus Lateralis and Gastrocnemius. Haematoxylin and eosin (H&E) staining on transverse sections of Vastus Lateralis from HU mice for 1-,2-,3- and 4-weeks showing time-dependent increasing extent of fibrotic area and reduction in muscle fiber diameters (**a**). Quantitative PCR (qPCR) showing mRNA expression levels of *Atrogin-1* in Vastus Lateralis (**b**–**e**), and Gastrocnemius (**f**–**i**) muscles of control mice kept in resting condition (Rest) and HU mice (Unload) at time points 1-2-3 and 4 weeks. The results showed a significant increase in *Atrogin-1* expression at 1 and 2 weeks in Vastus Lateralis (**b**, **c**) and at 2 weeks in Gastrocnemius (**g**) muscles. Shapiro-Wilk test and Mann-Whitney test were performed. Data are presented as box-and-whisker with median and interquartile ranges, from max to min, with all data points shown. *p < 0.05, **p < 0.01 vs Rest.

Hematoxylin and eosin (H&E) staining, performed on transverse sections of Vastus Lateralis from HU mice for 1-,2-,3- and 4-weeks, showed time-dependent increasing extent of fibrotic area and reduction in muscle fiber diameters (Fig. 1a). To investigate the time-dependent effect of unloading on disuse-induced muscle atrophy, we studied expression levels of *Atrogin-1*, the muscle-specific F-box protein, and Muscle ring finger 1 (*Murf-1)*, the ubiquitin ligase controlling proteasome-dependent degradation of muscle proteins, in Vastus Lateralis and Gastrocnemius of HU mice for 1-,2-,3- and 4-weeks.

Concerning the expression of *Atrogin-1* at mRNA level in Vastus Lateralis, we observed a significant 2-fold increase (*p = 0.016) at the 1-week of unloading (Fig. 1b) followed by an 11-fold increase (**p = 0.001) at 2-weeks (Fig. 1c) while no significant difference was found on 3rd and 4th week compared to the controls. The impact of unloading in the Gastrocnemius was slightly more delayed, as we found a significant 2-fold increase of *Atrogin-1* gene expression at 2-weeks (*p = 0.027) (Fig. 1g), without observing any modulation at the other time points compared to controls.

**Fig. 2:**
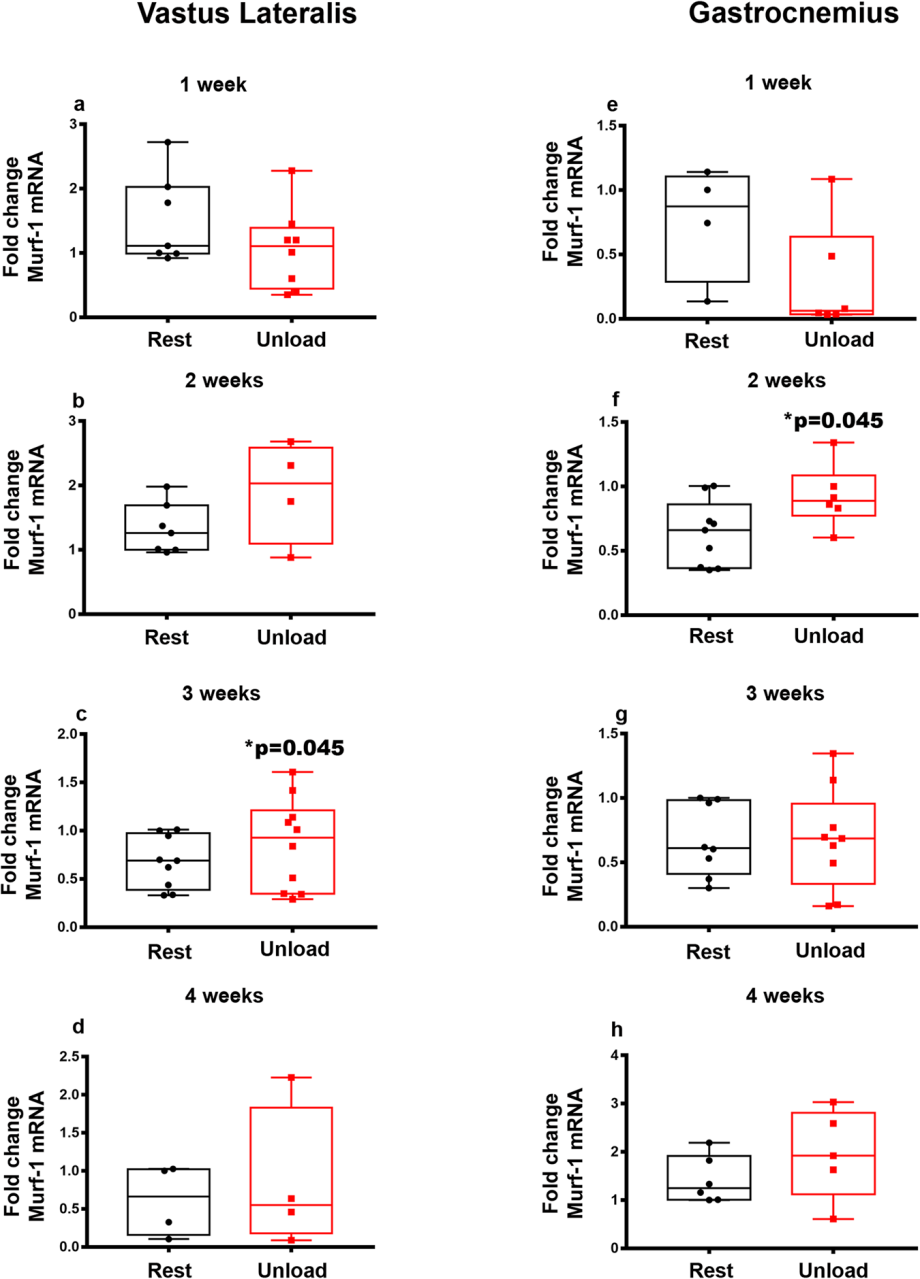
Effect of unloading on *Murf-1* mRNA expression in Vastus Lateralis and Gastrocnemius. Quantitative PCR (qPCR) showing mRNA expression levels of *Murf-1* in Vastus Lateralis (**a**–**d**), and Gastrocnemius (**e**–**h**) muscles of control mice kept in resting condition (Rest) and HU mice (Unload) at time points 1-2-3 and 4 weeks. The results showed a significant increase in *Murf-1* expression at 3 weeks in Vastus Lateralis (**c**) and 2 weeks in Gastrocnemius (**f**) muscles. Shapiro-Wilk test and Mann-Whitney test were performed. Data are presented as box-and-whisker with median and interquartile ranges, from max to min, with all data points shown. *p < 0.05 vs Rest.

We, then, analyzed the mRNA expression of *Murf-1* that increased only at 3-weeks of unloading in the Vastus Lateralis (*p = 0.045) (Fig. 2c) and only at 2-weeks in the Gastrocnemius (*p = 0.045) (Fig. 2f) with respect to controls.

**Fig. 3:**
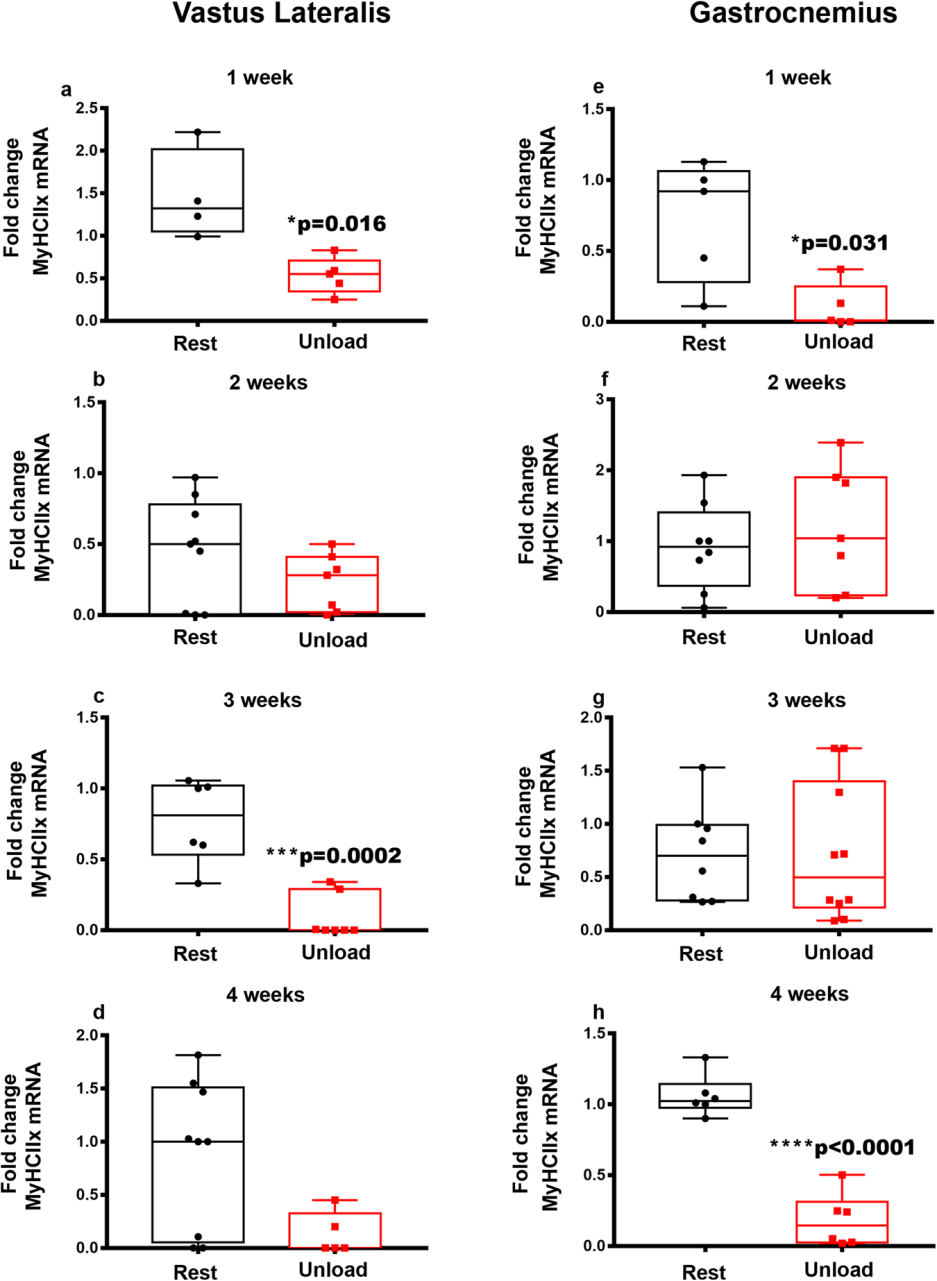
Effect of unloading on *MyHCIIx* myosin expression in Vastus Lateralis and Gastrocnemius. Quantitative PCR (qPCR) showing mRNA expression levels of *MyHCIIx* in Vastus Lateralis (**a**–**d**), and Gastrocnemius (**e**–**h**) muscles of control mice kept in resting condition (Rest) and HU mice (Unload) at time points 1-2-3 and 4 weeks. Analysis showed a down-regulation of *MyHCIIx* at 1 week of suspension in both Vastus Lateralis (**a**) and Gastrocnemius (**e**) muscles. The decrease of *MyHCIIx* was also observed at 3 and 4 weeks of suspension in Vastus Lateralis (**c**) and Gastrocnemius (**h**), respectively. Shapiro-Wilk test and Mann-Whitney test were performed. Data are presented as box-and-whisker with median and interquartile ranges, from max to min, with all data points shown. *p < 0.05, ***p < 0.001, ****p < 0.0001 vs Rest.

**Fig. 4:**
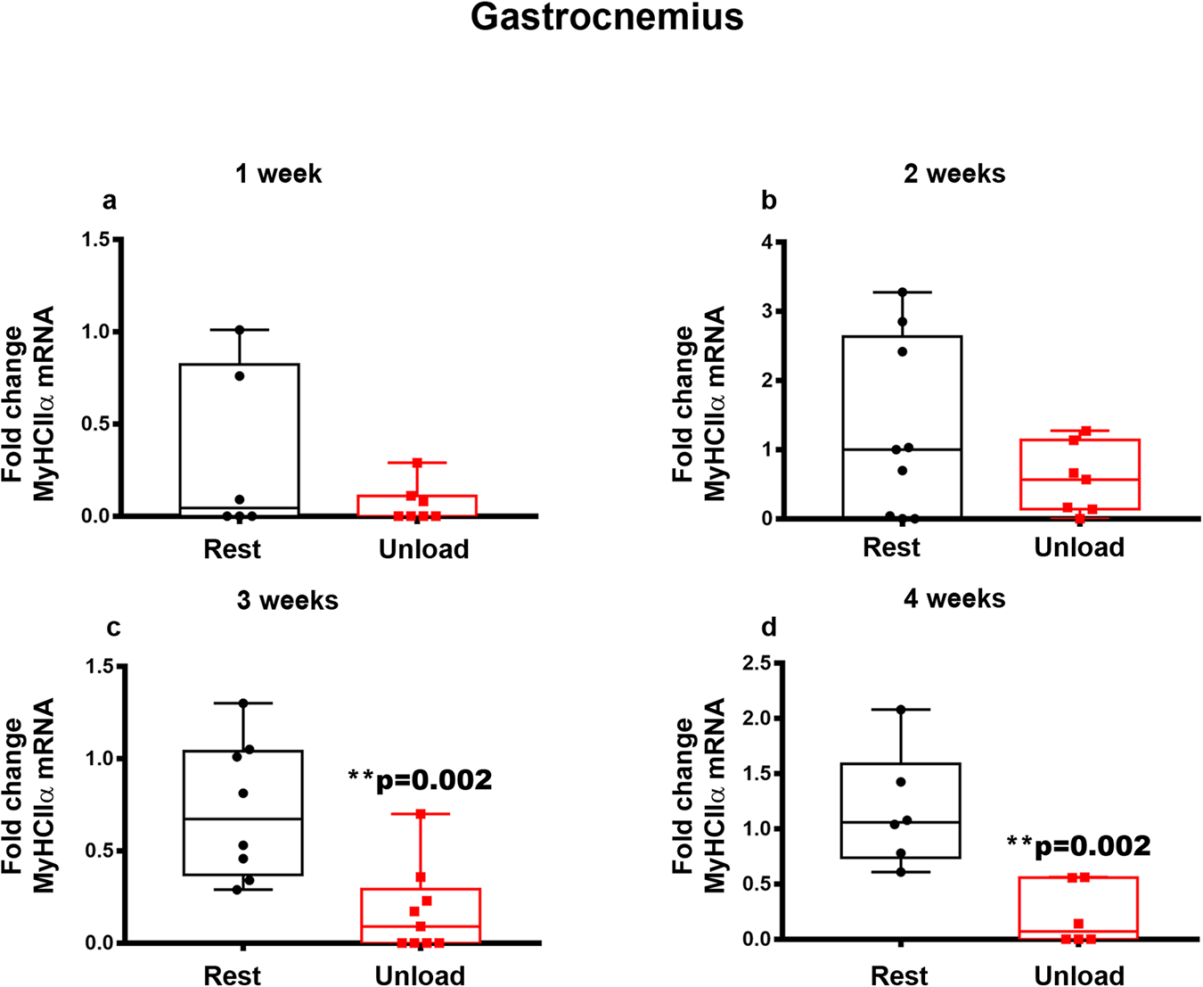
Effect of unloading on *MyHCIIα* myosin expression in Vastus Lateralis and Gastrocnemius. Quantitative PCR (qPCR) showing mRNA expression levels of *MyHCIIα* in Gastrocnemius (**a**–**d**) muscle of control mice kept in resting condition (Rest) and HU mice (Unload) at time points 1-2-3 and 4 weeks of suspension. Data showed a significant decrease of *MyHCIIα* in Gastrocnemius muscle after 3 (**c**) and 4 (**d**) weeks of suspension. Shapiro-Wilk test and Mann-Whitney test were performed. Data are presented as box-and-whisker with median and interquartile ranges, from max to min, with all data points shown. **p < 0.01 vs Rest.

Next, we studied the mRNA levels of myosin heavy chain (MyHC) isoforms in the same muscles and the same weeks of HU mice as described above. In the Vastus Lateralis, we observed that the gene expression of *MyHCIIx* mRNA was progressively reduced, starting with a significant reduction at 1-week (−64%, *p = 0.016) (Fig. 3a), more pronounced at 3-weeks (−88%, ***p = 0.0002) (Fig. 3c) and finally a decreasing trend at 4-weeks compared to control mice. Likewise, in the Gastrocnemius, unloading condition considerably decreased *MyHCIIx* mRNA expression at 1-week (−86%, *p = 0.031) (Fig. 3e), returned to basal levels at 2- and 3-weeks of unloading and then decreased again at 4-weeks (−83%, ****p < 0.0001) (Fig. 3h) compared to controls. Although in our previous study we observed a dramatic reduction of myosin type I (*MyHCI*) after 4-weeks unloading in Vastus Lateralis^12^, in the present time course study we did not observe changes of its expression at earlier time-points of unloading, nor of the other myosins examined (*MyHCIIα, MyHCIIβ*) in Vastus Lateralis (data not shown). However, we found a significant reduction in *MyHCIIα* mRNA levels in the Gastrocnemius of HU mice at 3- (−76%, **p = 0.002) (Fig. 4c) and 4-weeks (−86%, **p = 0.002) (Fig. 4d) of unloading compared to control conditions.

### FNDC5 gene expression in Vastus-Lateralis and Gastrocnemius muscles and Irisin serum levels are downregulated at 4 weeks of unloading

We have previously demonstrated that irisin stimulation for 4-weeks in HU mice preserved the number of muscle fibers in the Vastus Lateralis that co-express FNDC5 and ATPsynthase, thus indicating a possible correlation between the autocrine system FNDC5/Irisin and mitochondrial content in skeletal muscle^12^. Here we analyzed the effect of unloading over time on FNDC5 mRNA expression in Vastus lateralis and Gastrocnemius and, in parallel, we analyzed serum levels of endogenous irisin during the weeks of unloading.

**Fig. 5:**
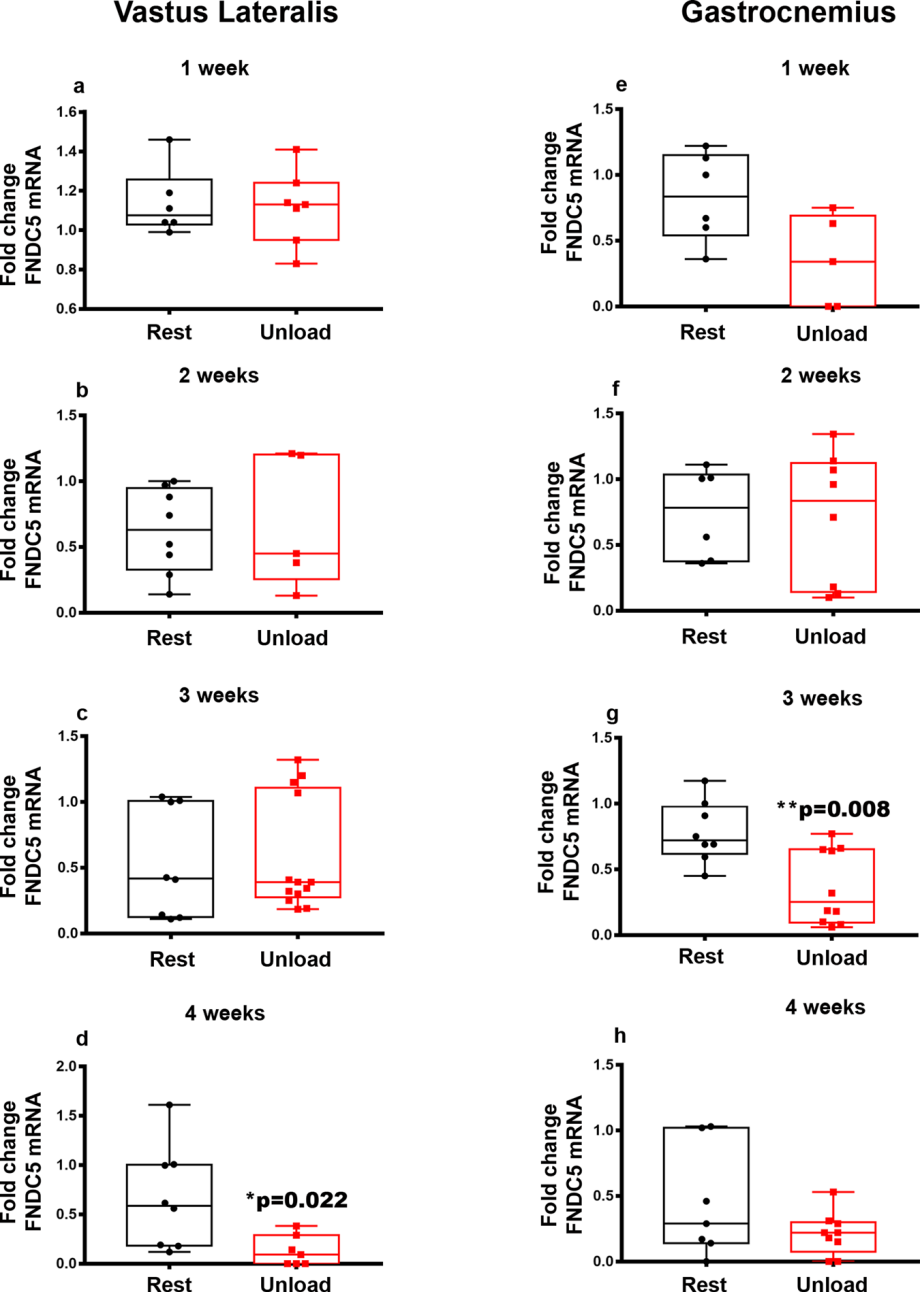
Effect of unloading on *FNDC5* mRNA expression in Vastus Lateralis and Gastrocnemius. Quantitative PCR (qPCR) showing mRNA expression levels of *FNDC5* in Vastus Lateralis (**a**–**d**) and Gastrocnemius (**e**–**h**) muscles of control mice kept in resting condition (Rest) and HU mice (Unload) at time points 1-2-3 and 4 weeks. Analysis showed a down-regulation of *FNDC5* expression at 4 weeks of suspension in Vastus Lateralis (**d**). In the Gastrocnemius, the downregulation of FNDC5 was observed at 3 weeks of suspension (**g**). Shapiro-Wilk test and Mann-Whitney test were performed. Data are presented as box-and-whisker with median and interquartile ranges, from max to min, with all data points shown. *p < 0.05, **p < 0.01 vs Rest.

*FNDC5* mRNA levels were significantly reduced at 4-weeks of unloading in the Vastus Lateralis, resulting 5-time lower than control mice (*p = 0.022) (Fig. 5d), while its reduction occurred earlier in the Gastrocnemius, as we observed 2-fold decrease at 3-weeks of unloading compared to the control mice (**p = 0.008) (Fig. 5g).

**Fig. 6:**
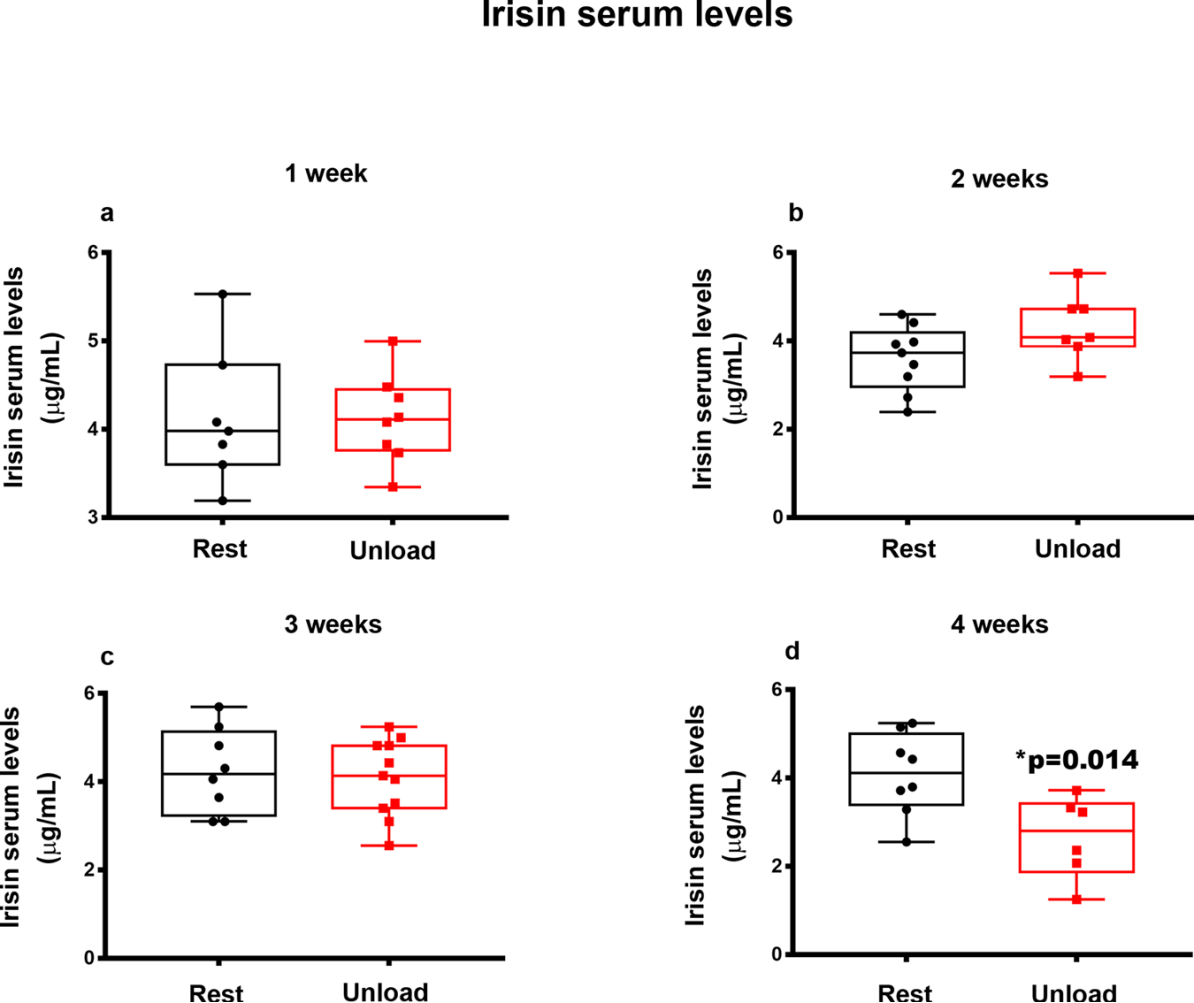
Effect of unloading on irisin serum levels. Figure 6 showed the trend in irisin serum levels of control mice kept in resting condition (Rest) and HU mice (Unload) for 1-2-3 and 4 weeks (**a**–**d**). Irisin serum levels were significantly reduced after 4 weeks of unloading compared to Rest mice (**d**). Shapiro-Wilk test and Mann-Whitney test were performed. Data are presented as box-and-whisker with median and interquartile ranges, from max to min, with all data points shown. *p < 0.05 vs Rest.

We did not observed any modulation of the circulating irisin levels until the 3rd week (Fig. 6a, b, c) but we detected a significant reduction (−35%, *p = 0.014) at the fourth week of unloading compared to the control mice (Fig. 6d).

### Unloading downregulates OPG and up-regulates the expression of senescent markers in bone tissue in vivo

**Fig. 7:**
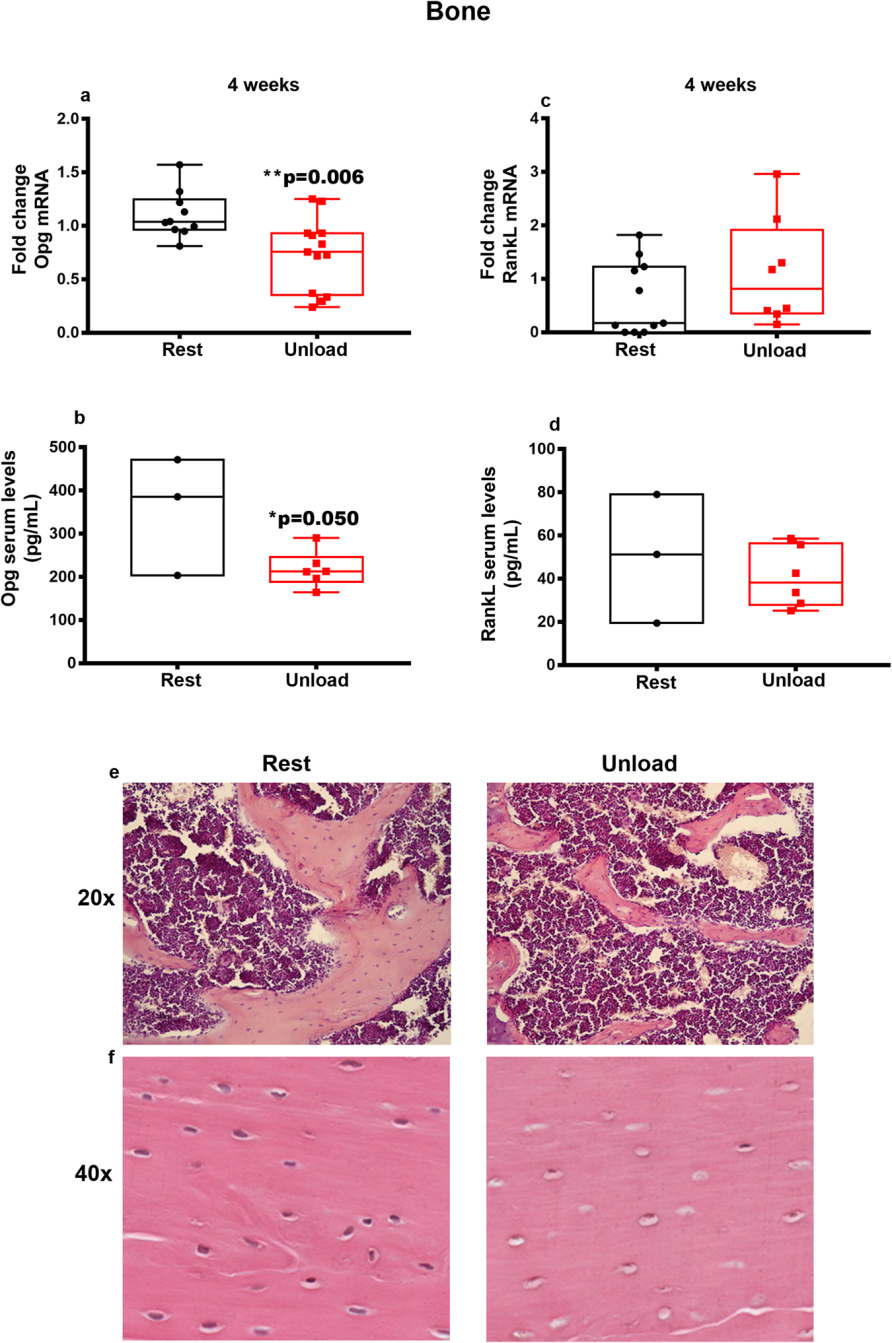
*Opg* and *RankL* mRNA expression in cortical bone under unloading condition. Quantitative PCR (qPCR) showing mRNA expression levels of *Opg* (**a**) and *RankL* (**c**) in cortical bone of control mice kept in resting condition (Rest) and HU mice (Unload) at 4 weeks. Data revealed a significant reduction in *Opg* expression levels in unloaded mice at 4 weeks (**a**), whereas there was an increasing but not significant trend of *RankL* in unloaded mice at 4 weeks (**c**). Measurements of OPG and RankL serum levels after 4 weeks of HU by Elisa Assays showing a significant reduction in OPG serum levels in HU mice compared to controls (**b**), but any significant change of RankL (**d**). Photomicrographs of hematoxylin and eosin (H&E)-stained sections of trabecular bone (**e**) (magnification 20x) and cortical bone (**f**) (magnification 40x) of femurs from Rest and Unload mice showing reduced thickness of trabeculae and increased number of apoptotic osteocytes in Unload mice compared to controls. Shapiro-Wilk test and Mann-Whitney test were performed. Data are presented as box-and-whisker with median and interquartile ranges, from max to min, with all data points shown. *p < 0.050; **p < 0.01 vs Rest.

One of the unloading-induced effects on bone tissue is the increase in bone resorption activity by osteoclasts controlled by the modulation of anti- and pro-osteoclastogenic cytokines i.e. Osteoprotegerin (OPG) and Receptor activator of nuclear factor kappa-B ligand (RANKL) respectively^16,17^. Here we show that in the cortical bone of HU mice, *OPG* and *RANKL* mRNA levels did not change during the first 2-weeks compared to controls (Supplemmentary Fig. 1a, b and d, e). At 3-weeks we observed that *OPG* gene levels tended to decrease while those of *RANKL* to increase (Supplementary Fig. 1c–f). At 4-weeks HU, *OPG* mRNA levels significantly decreased (−34%, **p = 0.006) (Fig. 7a) while the increasing trend in *RANKL* mRNA levels was not significant (Fig. 7c).

To corroborate the data obtained from gene expression analysis, we evaluated OPG and RankL serum levels after 4 weeks of HU. In line with Real Time-PCR results, we found a significant reduction in OPG serum levels in HU mice compared to controls (−38%, *p = 0.050) (Fig. 7b), whereas RankL serum levels showed no significant change (Fig. 7d). Moreover, in agreement with our previous works^12,18^, H&E staining on bone sections from control and HU mice at 4-week of unloading showed decreased thickness of trabeculae (Fig. 7e) and increasing number of empty lacunae indicating death of osteocytes (Fig. 7f).

**Fig. 8:**
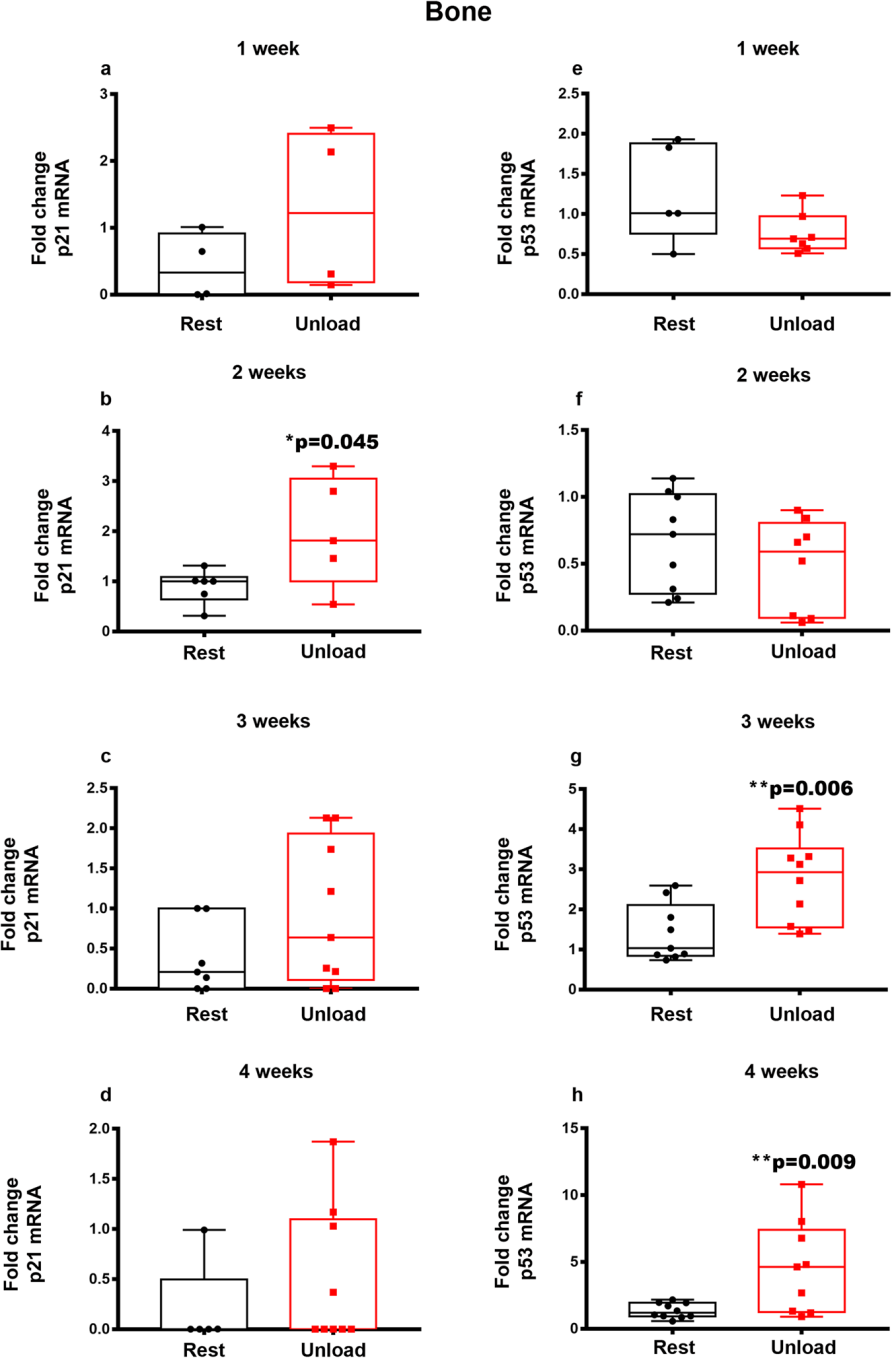
*p21* and *p53* mRNA expression in cortical bone under unloading condition. Quantitative PCR (qPCR) showing mRNA expression levels of *p21* (**a**–**d**) and *p53* (**e**–**h**) in cortical bone of control mice kept in resting condition (Rest) and HU mice (Unload) at time points 1-2-3 and 4 weeks. Data showed a significant increase of *p21* after 2 weeks (**b**) of suspension. In addition, we found an increase of *p53* expression at 3 (**g**) and 4 (**h**) weeks of suspension. Shapiro-Wilk test and Mann-Whitney test were performed. Data are presented as box-and-whisker with median and interquartile ranges, from max to min, with all data points shown. *p < 0.05, **p < 0.01 vs Unload.

In parallel, as microgravity induces cellular senescence^19,20^, we investigated the time- dependent effect of unloading on the gene expression of senescent markers (*p53*, *p21* and *p16* genes) in cortical bone of HU mice. We observed a 2-fold up-regulation (*p = 0.045) of *p21* mRNA levels (Fig. 8b) only in the second week of unloading, while *p53* gene levels increased by 2-fold (**p = 0.006) (Fig. 8g) and 3.5-fold (**p = 0.009) (Fig. 8h) at the third and fourth week of unloading, respectively, compared to controls.

### In vivo effects of irisin on musculoskeletal system during unloading

**Fig. 9:**
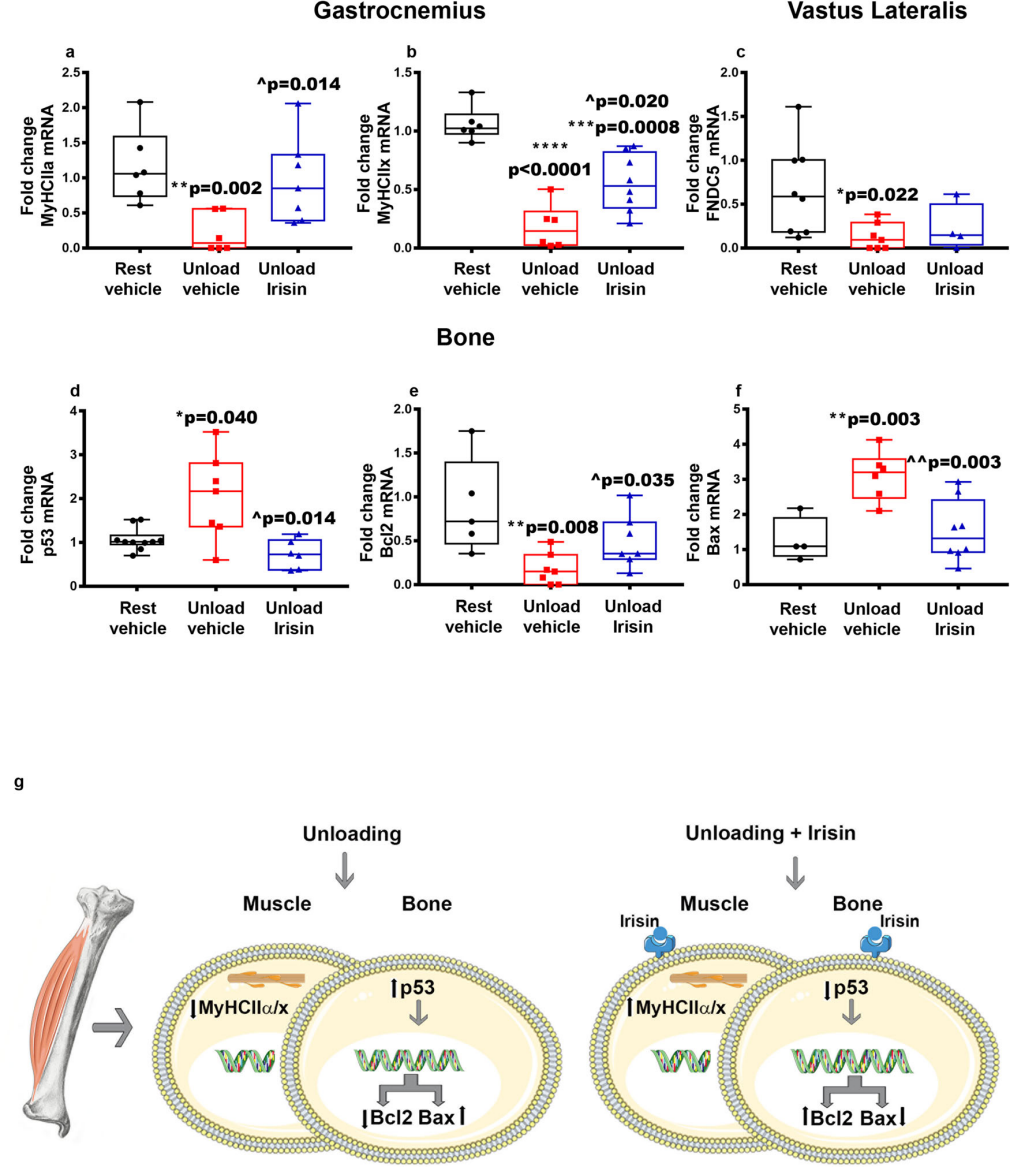
Effect of irisin on the musculoskeletal system of hind-limb suspended mice. Quantitative PCR (qPCR) showing mRNA expression levels of *MyHCIIα*, *MyHCIIx* in Gastrocnemius muscle (**a**, **b**), *FNDC5* in Vastus Lateralis muscle (**c**) and *p53*, *Bcl2* and *Bax* (**d**–**f**) in cortical bone of control mice kept in rest condition (Rest) and HU mice (Unload) treated with vehicle or irisin (100 μg/kg) once a week for 4 weeks. Data showed a significant reduction of *MyHCIIα* and *MyHCIIx* expression in unload mice treated with vehicle compared to controls (Rest), whereas irisin treatment restored *MyHCIIα* expression to control values (**a**). Albeit not returned to control levels, irisin treatment significantly increased the expression of *MyHCIIx* myosin compared to unload mice treated with vehicle (**b**). In Vastus Lateralis, irisin treatment was not effective in preventing *FNDC5* decline (**c**). The significant increase in *p53* expression in unload mice treated with vehicle was prevented by irisin treatment in unload mice (**d**). The unloaded-induced modulation of *Bcl2* and *Bax* were restored to control levels by irisin treatment in unload mice (**e**, **f**). Scheme summarizing the effects of unloading and irisin treatment on the expression of myosins in muscle and *p53*, *Bcl2* and *Bax* in cortical bone (**g**). Shapiro-Wilk test and Mann-Whitney test were performed. Data are presented as box-and-whisker with median and interquartile ranges, from max to min, with all data points shown. *p < 0.05, **p < 0.01, ****p < 0.0001 vs Rest and ^*p* < 0.05, ^^*p* < 0.001 vs Unload vehicle.

In our previous studies, we observed that the unloading condition decreased the expression of myosins type II in the Vastus Lateralis, in particular of the isoform IIX, while treatment with irisin restored myosin expression^12^. Therefore, in the present study we first focused on analyzing the effects of recombinant irisin treatment on unloading-induced modulations of myosin isoforms in the Gastrocnemius. Interestingly, we observed a reduction in *MyHCIIα* expression in HU mice (**p = 0.002), whereas in irisin-treated HU mice there was a significant increase of *MyHCIIα* expression compared to mice treated with vehicle (4.5-fold increase; ^*p* = 0.014). Notably, this increase restored *MyHCIIα* expression levels to those observed in rest mice (Fig. 9a). As already observed in our previous work in the Vastus Lateralis^12^, the unloading condition in the Gastrocnemius resulted in a reduction in *MyHCIIx* expression (****p < 0.0001), whereas irisin treatment led to a significant increase in *MyHCIIx* compared to vehicle-treated HU (3-fold increase; ^*p* = 0.020) (Fig. 9b). However, this increase did not completely restore values to the rest condition (−43%; ***p = 0.0008) (Fig. 9b).

As described above, the unloading condition caused a decrease in *FNDC5* in both Vastus Lateralis and Gastrocnemius. However, the irisin effect on *FNDC5* expression in the Vastus Lateralis (Fig. 9c) and in the Gastrocnemius (data not shown) was not statistically significant.

Based on the increase in *p53* mRNA levels in cortical bone and the reduction in serum irisin levels at 4 weeks of unloading described above, we next investigated whether irisin treatment, which restored bone loss in HU mice as previously showed^12^, could return *p53* levels to basal values. In parallel, as *p53* can trigger apoptosis in many cell types^21,22^, we further investigated if irisin could also modulate the levels of *Bcl2* and *Bax* mRNA involved in the apoptotic pathway. We first showed that in cortical bone the unloading-induced increase of *p53* respect to rest mice (+91%; *p = 0.040) (Fig. 9d), was restored in the irisin-treated HU mice returning to values of rest mice (−64%; ^*p* = 0.014; Unload vehicle vs Unload Irisin) (Fig. 9d). Moreover, *Bcl-2* expression, which was decreased in vehicle-treated HU (−80%; **p = 0.008) (Fig. 9e), significantly increased (+2.8-fold increase; ^*p* = 0.035) in irisin-treated HU mice returning to values of rest mice (Fig. 9e). On the contrary, expression of the pro-apoptotic *Bax*, which was up-regulated (+2.4-fold-increase; **p = 0.003) (Fig. 9f) in vehicle-treated HU mice, decreased in irisin-treated HU mice (−51%; ^^*p* = 0.003) and returned to the basal values of rest mice (Fig. 9f). These key genes involved in senescence and apoptosis have been also analyzed in skeletal muscles, but we did not find significant variations at the time points examined (data not shown).

### Irisin targets bone and muscle cells by supporting their differentiation and protecting them from senescence and apoptosis in vitro

To determine whether muscle and bone cells were the direct targets of irisin, we set up in vitro experiments with muscle cells, the C2C12 myoblast cell line, and bone cells, the MYLO-4 osteocyte and MC3T3 E1 osteoblast cell-line, to evaluate gene levels of their main differentiation markers and the expression of genes implicated in cell senescence and apoptosis.

**Fig. 10:**
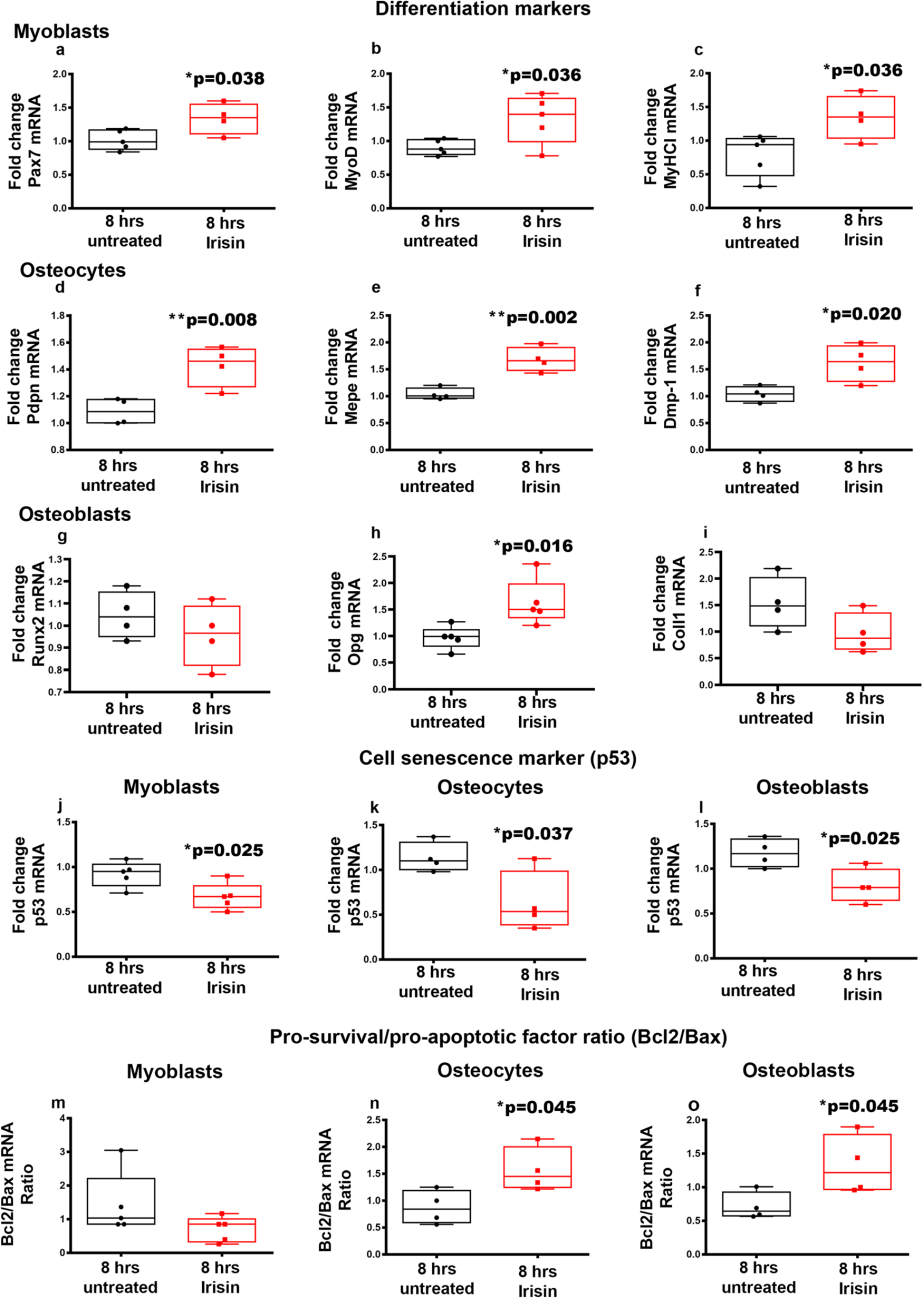
Effect of irisin on myoblast, osteocyte and osteoblast cell lines. Quantitative PCR (qPCR) showing mRNA expression levels of genes involved in myoblast, osteocyte, and osteoblast differentiation. In myoblasts (C2C12) treated for 8 h (hrs) with irisin (100 ng/mL), the data showed an up-regulation of Pax7 (**a**), MyoD (**b**) and MyHCI (**c**), key regulators of myoblast differentiation process. Likewise, irisin treatment increased the expression of Pdpn (**d**), Mepe (**e**) and Dmp-1 (**f**) in the osteocyte line MLO-Y4. In osteoblast (MC3T3) irisin treatment increased the expression of Opg (**h**) that has an anti-resorptive effect on bone but did not change the expression of Runt-related transcription factor 2 (*RunX2*) (**g**) and Collagen type I (*Coll1*) (**i**). Quantitative PCR (qPCR) showing mRNA expression levels of genes involved in senescence and apoptosis in myoblasts, osteocytes, and osteoblasts. Following 8 h treatment with irisin (100 ng/mL), the expression of the senescence marker gene p53 was down-regulated in myoblasts (C2C12) (**j**), osteocytes (MLO-Y4) (**k**) and osteoblasts (MC3T3) (**l**). In addition, treatment with irisin for 8 h increased the Bcl2/Bax pro-survival ratio in both osteocytes (**n**) and osteoblasts (**o**), whereas in C2C12 myoblasts this ratio showed no significant difference compared to untreated cells (**m**). Shapiro-Wilk test and Mann-Whitney test were performed. Data are presented as box-and-whisker with median and interquartile ranges, from max to min, with all data points shown. *p < 0.05, **p < 0.01 vs 8 h untreated.

First, we demonstrated that 8-h irisin stimulation increased expression of genes implicated in both the early and late stages of myogenesis, namely the paired box protein 7 (*Pax7*) (+31%; *p = 0.038), (Fig. 10a) and the myoblast determination protein 1 (*MyoD*) (+47%; *p = 0.036), (Fig. 10b), and enhanced the expression of *MyHCI* (+70%; *p = 0.036) (Fig. 10c). Next, we proved that 8-h irisin stimulation induced the expression of osteocyte selective genes, such as podoplanin (*Pdpn*) (+31%; **p = 0.008), (Fig. 10d), matrix extracellular phospho-glycoprotein (*Mepe*) (+61%; **p = 0.002), (Fig. 10e) and matrix extracellular phospho-glycoprotein 1 (*Dmp1*) (+55%; *p = 0.020), (Fig. 10f) in MLO-Y4 osteocytes. In MC3T3 E1 osteoblast cell line, 8-h irisin stimulation increased osteoprotegerin (*Opg*) expression (+60%; *p = 0.016), (Fig. 10h), but did not change the expression of Runt-related transcription factor 2 (*RunX2*) (Fig. 10g) and Collagen type I (*Coll1*) (Fig. 10i).

In studying the effect of irisin on senescence and apoptosis in both muscle and bone cells, we demonstrated a decrease of *p53* mRNA levels in irisin-treated myoblasts, (−27%, *p = 0.025), (Fig. 10j), and bone cells, specifically osteocytes (−44%, *p = 0.037), (Fig. 10k), and osteoblasts (−54%; *p = 0.025), (Fig. 10l). Finally, we did not observe any change of the *Bcl2/Bax* ratio (Fig. 10m) in myoblast, but we found an increased *Bcl-2/Bax* ratio in osteocytes (+79%; *p = 0.030) (Fig. 10n) and in osteoblasts (+85%; *p = 0.045), (Fig.10o), indicating an anti-apoptotic effect of irisin on bone cell lines.

## Discussion

In the present study, by investigating the time-dependent effect of unloading on musculoskeletal system, we show that in HU mice weightlessness-induced changes target muscle tissue before bone tissue, and irisin levels are critical in regulating bone senescence and apoptosis both in vivo and in vitro. Skeletal muscle wasting is well documented in different disuse-induced animal models, and it is established that the decline of muscle mass arises in the first two weeks of unloading although the level of mass loss depends on the muscle type and the unloading degree^23^. In keeping with this, we have previously shown loss of muscle mass and reduction of cross section fiber in the Vastus Lateralis of HU mice for 4 weeks^12^. Because atrophy and imbalance of protein synthesis contribute to muscle mass loss, in this study we investigated the time-course effect of unloading on the gene expression of atrophy markers and myosins in Vastus Lateralis and in Gastrocnemius muscles of HU mice starting from 1 up to 4 weeks of unloading. The Vastus Lateralis and Gastrocnemius muscle fibers of these mice rapidly became atrophic, as, from the first week of unloading, we detected the up-regulation of *Atrogin-1* and *Murf1*, and a down-regulation of intermediate and fast myosin heavy chain. Specifically, in the Vastus Lateralis, we found an increased expression of *Atrogin-1*, in the first and second week of unloading, while *Murf-1* increased in the third week, thus indicating a later response of this gene to unloading. Gastrocnemius was also affected by unloading with the modulation of *Atrogin-1* and *Murf-1* at the second week. Activation of ubiquitin proteasome pathways, implicated in the breakdown of myofibrillar proteins in disuse, requires transcriptionally dependent up-regulation of these atrogens, whose induction precedes muscle atrophy^24^. In most studies, performed on the soleus of HU mice, an antigravity muscle preferentially affected by unloading, an early (3 days of unloading) and transient increase in *Atrogin-1* and *Murf-1* mRNA has been observed, which returned to basal levels after 1 week of unloading^23,25,26^. In the present study we show that, although later but more persistently over time than in soleus, up-regulation of atrogen genes also occurs in Vastus Lateralis and Gastrocnemius, providing new readouts for translational research on the HU mouse model.

One of the key events that occurs immediately after triggering ubiquitin proteasome pathway is the change in myosin phenotype. Studies performed on astronauts after 6 months aboard the International Space Station demonstrated a slow-to-fast fiber type transition in the Gastrocnemius and Soleus muscles^27^. A reduction in *MyHC I* mRNA expression was observed in rat soleus as early as 1 day following HU^28,29^. Lomosova et al. found that the expression of *MyHC IIa* mRNA in rat Soleus decreased at day 3 of HU returning to control levels at day 7, in parallel with an increase in the expression of *MyHC IIx* and *MyHC IIb* from day 3 to day 14 of HU^30^.

Unlike what was extensively showed in the muscle Soleus, in Vastus Lateralis and Gastrocnemius we didn’t observe any time-dependent change of slow myosin heavy chains during unloading, whereas intermediate and fast myosins were modulated. Basically, the Soleus muscle has a higher proportion of slow twitch fibers (70%) than Gastrocnemius and Vastus Lateralis which contain a percentage of slow twitch fibers of about 50% and 32%, respectively, and therefore the latter muscles are more prone to modulation of intermediate and fast myosins^31^. Specifically, we found that the intermediate *MyHCIIα* decreased only in Gastrocnemius in the last two weeks of unloading, whereas the fast *MyHCIIX* was modulated in both muscles analyzed. Notably, in Vastus Lateralis the expression of *MyHCIIX* had an “up and down” modulation starting from week 1 of unloading, with a decrease in myosin at week 1 and 3 of suspension, while it returned to basal level at week 2 and 4. In Gastrocnemius, the effect of unloading was different: *MyHCIIX* decreased at the first and fourth week while it returned to the basal level during the second and third week. Thus, our data demonstrate that, during unloading, the myosin phenotype is very dynamic and differently dependent on muscle type and duration of unloading by responding with alternating phases of myosin modulation and phases of myosin rearrangement after initial atrophy. Regarding the possible role of atrophy genes in the modulation of myosins during the time-course of unloading, we hypothesized that the highest peak of *Atrogin-1* in Vastus Lateralis and of *Murf-1* in Gastrocnemius might be responsible for the decrease of *MyHCIIa* and *MyHCIIx* during unloading (Supplementary Fig. 2).

In response to muscle contraction, skeletal muscle secretes numerous soluble molecules known as myokines^3,4^. Therefore, although not yet widely demonstrated, it has been hypothesized that reduced muscle function may negatively impact the release of these molecules^32^. Moreover, in consideration of our findings showing that the myokine irisin has an anabolic effect on the muscle^12^, we studied the time dependent effect on the irisin precursor *Fndc5* during unloading, to investigate whether the catabolic effect of unloading could influence its expression. In Vastus Lateralis we observed its significant reduction at the fourth week of unloading, while in Gastrocnemius the reduction of *Fndc5* occurred earlier, at the third week, demonstrating that the time dependent effect on this gene is different in these muscles. Because of these alterations in the irisin precursor *Fndc5*, serum irisin levels decreased significantly at 4-week of unloading. A possible explanation for the late effect on *Fndc5*/irisin reduction at 4 weeks in unloaded mice, as opposed to the peak of *Atrogin-1* occurring as early as the 1-week HU, is that the myokine decline might not be dependent on atrophy per se, but on a reduction in muscle contraction that affects mice at an advanced stage of the unloading period, as also discernible from the reduction in the expression of contractile proteins (i.e., myosins). In addition, although cortisol was not measured, which may be a limitation of our study, we cannot exclude that the stress caused by hind limb unloading may have induced the expression of glucocorticoids^33^, which in turn decreased the expression of *Fndc5* in muscle^34^.

To explore whether the effects of unloading on muscle were linked to the reduction in circulating levels of irisin, we treated unloaded mice with exogenous irisin for four weeks. Our data showed that irisin induced in Gastrocnemius a total recovery of *MyHCIIα* expression, while it was partial for the unloading-induced decrease of *MyHCIIX*. This finding confirms what was previously observed in Vastus Lateralis by our group, where irisin inhibited the decrease in *MyHCIIX* expression caused by unloading^12^ and support the anti-atrophic effect of irisin in muscles.

The positive effect of irisin on muscle is further supported by in vitro data on C2C12 cells treated with recombinant irisin. It might be that irisin enhances myogenesis and induces hypertrophy by increasing *Pax7*, expressed in the early stages of myogenesis, *MyoD*, expressed in the later stages in myoblast, and *MyHC-I*, expressed in myotubes. These data support the hypothesis that irisin might not only potentially promote increased proliferation of myoblast cell and driving them towards differentiation, but also by enhancing the fusion of myoblasts in myotubes. Thus, our data reveal how irisin may contribute to accelerate the process of myogenesis toward myotubes formation from the earliest stages of myogenesis, as previously showed by other authors^35^. Moreover, the increase in cell differentiation accompanied by a decrease in *p53* expression in C2C12 treated with irisin, highlights the irisin potential to preserve muscle cell from senescence.

Unloading determines alterations on bone tissue at a later stage than muscle^36^. Accordingly, *Opg* downregulation at the fourth week of unloading contributes to support the osteoclast formation and their resorptive activity, which leads to the bone loss that follows muscle atrophy occurring within 2 weeks^37–39^. In addition, the senescence marker genes are affected by unloading in bone. *p21* increased in the second week of unloading, while *p53* increased starting from the third week, thus suggesting that unloading promotes acquisition of a senescent phenotype.

However, due to the involvement of p53 in the induction of apoptosis^40,41^, the unloading-induced up-regulation of its expression suggests that hypomobility also promotes apoptosis in bone in addition to senescence. Both effects are inhibited by exogenous irisin during 4 weeks of unloading, as shown by the reversal of up-regulation of the senescence marker gene p53 and the pro-apoptotic gene Bax induced by disuse. These cellular responses, in addition to reduced serum irisin levels, may be critically implicated in bone damage. In addition, these findings are also supported by in vitro data showing irisin effects on senescence and apoptosis in bone cells. Irisin decreased *p53* expression and increased the *Bcl-2/Bax* ratio in both osteoblast and osteocyte cells. Moreover, irisin treatment increased mRNA levels of osteocyte marker genes such as *Pdpn*, *Dmp-1* and *Mepe*, suggesting that this myokine has a strong effect on osteocytes not only protecting them from the apoptosis but also increasing their differentiation.

Overall, our data reveal the time-dependent impact of unloading on gene expression in muscle and bone and which of these impairments can be counteracted by irisin treatment. Further studies are encouraged to understand whether irisin could be a promising clinical strategy for the prevention and treatment of disuse-induced disorders affecting muscle and bone in bedridden or elderly patients. Moreover, an irisin-based therapy could also represent a potential countermeasure for astronauts who are exposed to microgravity during their space flight missions.

## Methods

### Animal models

2-months-old mice C57BL6 male and female mice (*n* = 64; 34 male mice and 30 female mice) were randomly assigned to two groups: mice kept in control condition (Rest) and hind-limb suspended mice (Unload). Each group was further divided into 4 subgroups: Rest (*n* = 6) and Unload (*n* = 7) for 1 week; Rest (*n* = 7) and Unload (*n* = 8) for 2 weeks; Rest (*n* = 8) and Unload (*n* = 13) for 3 weeks; Rest (*n* = 8) and Unload (*n* = 7) for 4 weeks. In addition to the 4-week time point, we added a further group of mice, Unload Irisin (*n* = 8), suspended and treated for 4 weeks with rec-Irisin (Adipogen) 100 μg/kg once a week for 4 weeks. Hind-limb suspended mice were subjected to the tail suspension procedure, according to recommendations by Wronski and Morey-Holton^42^. Hind-limb suspended mice were adjusted to prevent any contact of the hind limbs with the cage floor, resulting in approximately a 30° head-down tilt. The forelimbs of the animals were in contact with the cage bottom, allowing the mice fill access to the entire cage. Each mouse was singly housed, maintained under standard conditions on a 12/12 h light/dark cycle and with access to water and regular diet ad libitum (Harlan Teklad 2019, SDS, England).

Mice were weighed once a week and at the end of the experimental procedures were euthanized and their tissues were surgically excised. At the sacrifice, using appropriate techniques, the muscles gastrocnemius and vastus lateralis were harvested and stored at −80 °C until analysis. The left tibiae were subjected to bone marrow flushing and stored at −80 °C. This animal interventional study is in accordance with the European Law Implementation of Directive 2010/63/EU and all experimental protocols were reviewed and approved by the Veterinary Department of the Italian Ministry of Health (Project 522-2016PR). Experimental procedures have been carried out following the standard biosecurity and the institutional safety procedures. Investigators were blinded to the group allocation. Power analysis: for α of 0.05 and *p* < 0.05; 4–13 mice/group. Sample sizes were chosen based on pilot studies and prior related work.

### Histological analysis of muscle

Vastus lateralis muscles were excised from the quadriceps, fixed, and embedded in paraffin. 5 µm thick histological sections were cut and stained with hematoxylin and eosin (H&E). All observations were performed with a Nikon Eclipse 80i light microscope (Nikon) at magnification of 20x by using the NIS-Element BR 4.10.00 software.

### Histological analysis of bone

Freshly dissected femurs were immediately fixed in ice-cold 4% paraformaldehyde solution for 4 h. After decalcification, performed with 0.5 M ethylenediaminetetraacetic acid (EDTA) at 4 °C, bones were immersed into 20% sucrose and 2% polyvinylpyrrolidone (PVP) solution for 24 h. Femurs were then embedded and frozen in optimal cutting temperature (OCT) compound (VWR Chemicals). For histological analyses, sections were generated by using a SLEE MEV Semi-Automatic Cryostat (SLEE medical GmbH). 5 µm thick histological sections were cut and stained with hematoxylin and eosin (H&E). All observations were performed with a Nikon Eclipse 80i light microscope (Nikon) at magnification of 20x and 40x by using the NIS-Element BR 4.10.00 software.

### Cell cultures

Mouse preosteoblast MC3T3-E1 (ATCC), Mouse osteocytes MLO-Y4 (Kerafast, Inc., Boston, MA, USA) and Mouse myoblasts C2C12 (ATCC) were used for in vitro experiments in this study. MC3T3 cell line was plated at 10 × 10^3^ cells/cm^2^ and cultured in Minimum Essential Medium Eagle - Alpha Modification (α-mem) (Gibco; Thermo-Fisher, Waltham, MA) with 10% fetal bovine serum (Gibco; Thermo-Fisher) until they reached confluence in a humidified atmosphere (37 °C, 5% CO2) (Hera cell 150; ThemoFisher). Upon confluence, to induce differentiation and mineralization for preosteoblast MC3T3-E1 cells, we cultured them with α-mem medium supplemented with 5 μg/mL ascorbic acid and 10^−2^ M β-glycerophosphate for 12 days. Before culturing MLO-Y4 osteocytes we coated a 100-mm × 20-mm plates (Corning, Corning, NY, USA) with collagen (0.15 mg/mL) (Sigma-Aldrich, St. Louis, MO, USA) for 1 h under a sterile tissue culture hood at room temperature. After coating, plates were washed with PBS (Gibco, Thermo-Fisher, Waltham, MA, USA). Then cells were plated at 10 × 10^3^ cells/cm^2^ and cultured in α-MEM (Gibco, Thermo-Fisher, Waltham, MA, USA) with 10% of fetal bovine serum (FBS) (Gibco, Thermo-Fisher, Waltham, MA, USA) to maintain differentiation in a humidified atmosphere (37 °C, 5% CO2), (Hera cell 150; Themo-Fisher, Waltham, MA, USA) until reaching the confluence.

Mouse myoblasts C2C12 were plated at 10 × 10^3^ cells/cm^2^ and cultured in α-MEM (Gibco, Thermo-Fisher, Waltham, Massachusetts, United States) with 10% of fetal bovine serum (FBS), (Gibco, Thermo-Fisher, Waltham, Massachusetts, United States) until they reached confluence in a humidified atmosphere (37 °C, 5% CO2), (Hera cell 150, Themo-Fisher, Waltham, Massachusetts, United States). Upon confluence, we induced the C2C12 differentiation by replacing the growth medium with the differentiating medium, α-MEM with 2% of FBS (Gibco, Thermo-Fisher, Waltham, Massachusetts, United States) to stimulate myotube formation. The cells were grown in differentiating medium for 10 days.

All cell line were treated with irisin (100 ng/mL) in the last 8 h of the culture.

### Real time-PCR

Bone and muscle samples were homogenized with ultra-turrax T8 (Ika, Staufen im Breisgau, Germany). Total RNA from mouse tissues and cells was extracted using spin columns (RNeasy, Qiagen) according to the manufacturer’s instructions. DNase I treatment was performed to remove genomic DNA contamination (Qiagen) and RNA integrity was assessed on agarose gels. Reverse transcription was performed using iScript Reverse Transcription Supermix (Bio-Rad, Hercules, California, US). The resulting cDNA (125 ng for bone and 1μg for muscle) was subjected to quantitative PCR (qPCR) using the SsoFast EvaGreen Supermix (Bio-Rad) on Bio-Rad CFX96 Real-Time System (Bio-Rad) for 40 cycles (denaturation 95 °C for 5 s; annealing/extension 60 °C 10 s) after an initial 30 s step for enzyme activation at 95 °C. To confirm the specificity of amplification products, melting curve was performed between 65–96 °C, with 0.5 °C incrementing every 10 s. Primers were designed by using Primer Blast (https://www.ncbi.nlm.nih.gov/tools/primer-blast/). We chose Gapdh as housekeeping gene because is stably expressed in bone and muscle. All primers span an exon-exon junction. Each transcript was assayed in triplicate and quantitative measures were obtained using the ΔΔCT method and expressed as a fold change compared to control. Primers sequences were indicated in Supplementary Table 1.

### Elisa assay

Mice blood samples were collected at the end of each end points. Blood was collected into serum tubes and allowed to clot for 30 min at room temperature before centrifugation for 15 min at 1000 × *g*. After centrifuging, the samples were aliquoted and stored at −80 °C until analysis.

Irisin serum concentrations were detected using a competitive ELISA kit (Adipogen, Liestal, Switzerland) with an inter-assay coefficient of variation ≤6.9%. The ELISA kit allows the largest range of measurement (0.001–5 µg/ml) and is the most sensitive (0.001 µg/ml). The ELISA kit includes a polyclonal antibody recognizing naïve Irisin and recombinant Irisin under competition in Irisin-coated plates. In accordance with the manufacturer’s instructions, the Irisin competitive ELISA kit (Adipogen) is specific for the measurement of natural and recombinant irisin in human samples. It also works in mouse, rat and monkey biological samples. It does not cross-react with FNDC4, human adiponectin, human Nampt, human RBP4, human clusterin, human leptin, human vaspin, human GPX3, human resistin, human ACE2, human lipocalin-2, human ANGPTL3, human ANGPTL6, human DNER, human DLK1, human carleticulin, human IL-33, mouse Nampt, mouse clusterin, mouse vaspin and mouse resistin.

Osteoprotegerin/TNFRSF11B serum levels were detected using Quantikine ELISA Immunoassay kit (R&D System, Inc.,Minneapolis, US). This assay employs the quantitative sandwich enzyme immunoassay technique. A monoclonal antibody specific for mouse OPG has been pre-coated onto a microplate. Standards, control and samples are pipetted into the wells and any mouse OPG present is bound by the immobilized antibody. Regarding the sensitivity of the kit, in accordance with the manufacture istructions, the minimum detectable dose (MDD) of mouse OPG ranged from 1.0–6.9 pg/mL. The mean MDD was 2.8 pg/mL. This assay recognizes natural and recombinant mouse OPG and no significant cross-reactivity and interference was observed.

TRANCE/RANKL/TNFSF11 concentrations were detected using Quantikine ELISA Immunoassay (R&D System, Inc., Minneapolis, US). This kit is based on the quantitative sandwich enzyme immunoassay technique. An affinity purified polyclonal antibody specific for mouse TRANCE has been pre-coated onto a microplate. Standards, control and sample are pipetted into the wells and the immobilized antibody binds any TRANCE present. The minimum detectable dose (MDD) of mouse TRANCE is typically less than 5.0 pg/mL. This assay recognizes free natural and recombinant mouse TRANCE. No significant cross-reactivity or interference was observed.

### Statistical analysis

All data were subjected to the Normality test (Shapiro-Wilk) and the significance was calculated by Mann-Whitney test that is more appropriate for not normally distributed data and for small sample sizes. The results were considered statistically significant for *p* values ≤ 0.05. Data are presented as box-and-whisker plots, with median and interquartile ranges, from max to min, with all data points shown. GraphPad Prism 7.0 were used to analyze data.

### Reporting summary

Further information on research design is available in the Nature Research Reporting Summary linked to this article.

## Supplementary information

Supplentary Information Files

Reporting Summary

## Data Availability

The data that support the findings of this study are available on request from the corresponding author.
