# Supplementary material for: Time-dependent unloading effects on muscle and bone and involvement of FNDC5/irisin axis
Source: NPJ Microgravity. 2023 Jan 19;9:4. doi: 10.1038/s41526-023-00251-w (PMC9852594; doi:10.1038/s41526-023-00251-w)
Supplement: Supplementary file 1 — Supplentary Information Files [file 41526_2023_251_MOESM1_ESM.pdf]

Opg and *RankL* mRNA expression in cortical bone under unloading condition

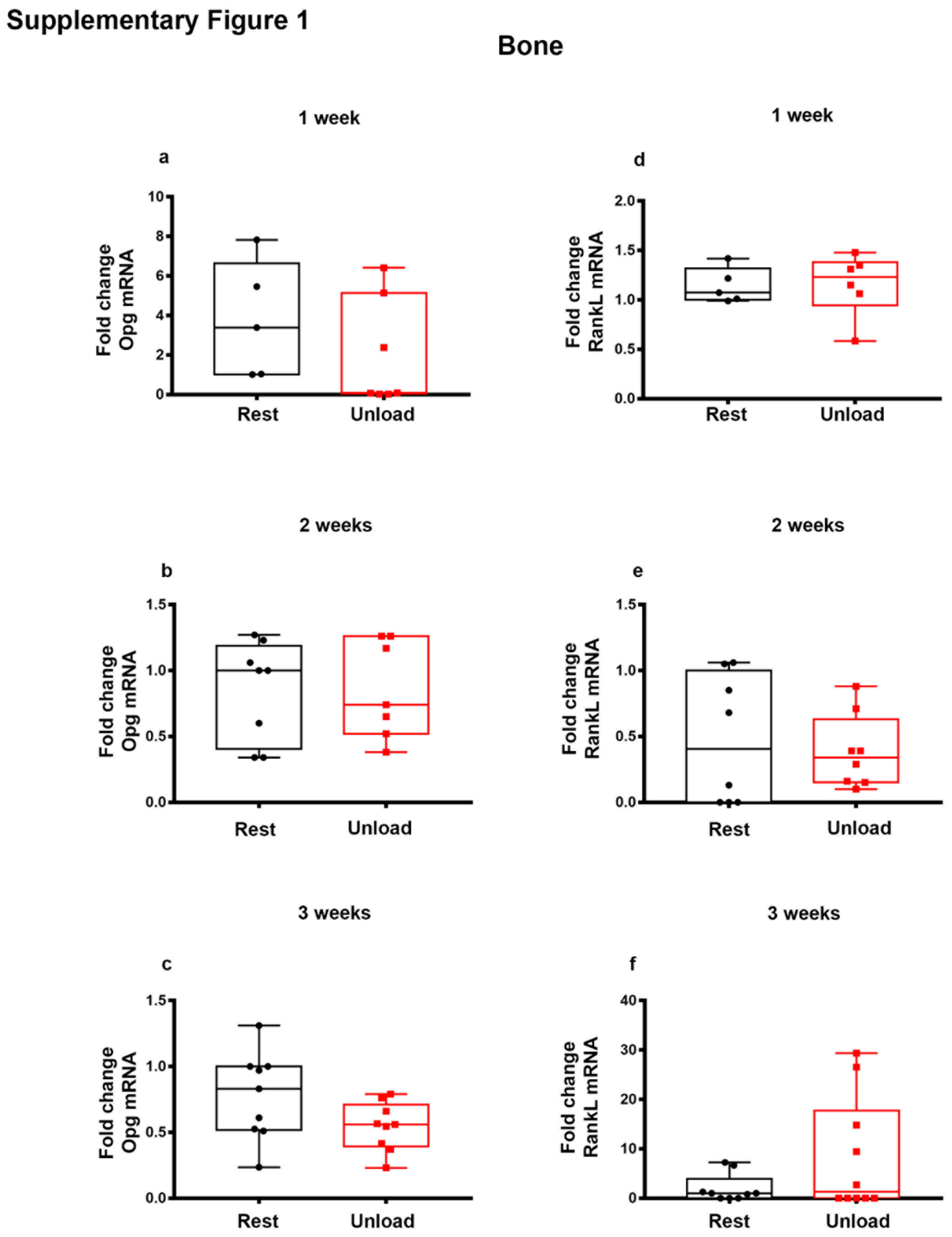

Supplementary Figure S1. Quantitative PCR (qPCR) showing mRNA expression levels of *Opg* (a-b-c) and *RankL* (d-e-f) in cortical bone of control mice kept in resting condition (Rest) and HU mice (Unload) at time points 1-2-3 weeks. Shapiro-Wilk test, and Student t-test or Mann-Whitney test were performed. Data are presented as box-and-whisker with median and interquartile ranges, from max to min, with all data points shown.

Overlapping trends of *Atrogin-1* and *Murf-1* expressions with those of *MyHCIIa* and *MyHCIIx* examined during the unloading time-course

Supplementary Figure 2

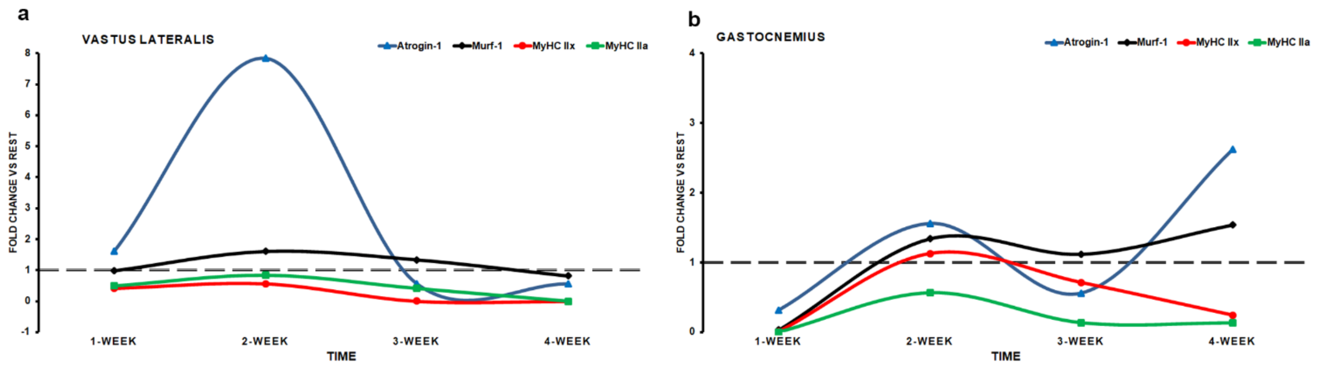

Supplementary Figure S2. Data points were calculated for each gene as fold change of the median of Unload versus Rest mice.

Supplementary Table 1

Sequence, accession number (NM_) and product length for each primer

**Gene name and ID**	Forward (5´–3´) and Template	Reverse (5´–3´) and Template	product length
GAPDH XM_036165840.1	TGCGACTTCAACAGCAACTC	CTTGCTCAGTGTCCTTGCTG	200
FNDC5 XM_006503212.5	GTGCTGACTATTGTTGTGGTCC	ATCATATCTTGCTGCGGAGGAG	169
Atrogin-1 NM_026346.3	TGAGCGACCTCAGCAGTTAC	GCGCTCCTTCGTACTTCCTT	182
Murf-1 NM_001369245.1	ACCTTCCTCTCAAGTGCCAAG	TCCCAAAGTCAATGGCCCTC	157
MyHCIIα NM_001039545.2	ATAAAAGAGTCCCGAACGAGGC	GCTGAACTCACAGACCCTTAC	110
MyHCIIx NM_030679.2	AAGTTGCATCCCTAAAGGCAG	TGTTCTGAGCCTCGATTCGC	145
OPG XM_036159230.1	GACCACCTTTATACGGACAG	CTCACACTCACACACTCG	126
RankL NM_011613.3	CCCATCGGGTTCCCATAAAGT	CCCGATGTTTCATGATGCCG	162
p53 XM_030245923.2	TCTTATCCGGGTGGAAGGAAA	GGCGAAAAGTCTGCCTGTCTT	62
p21 NM_001111099.2	GCAGAATAAAAGGTGCCACAGG	AAAGTTCCACCGTTCTCGGG	176
Bax NM_007527.3	AGATGAACTGGACAGCAATATGG	GCAAAGTAGAAGAGGGCAACC	150
Bcl2 NM_009741.5	GGACTTGAAGTGCCATTGGT	CAGGCTGGAAGGAGAAGATG	205
Collagen I NM_007742.4	GGCTCCTGCTCCTCTTAG	ACAGTCCAGTTCTTCATTGC	194
RunX2 NM_001145920.2	TCGGAGAGGTACCAGATGGG	TGAAACTCTTGCCTCGTCCG	159
